# Factors Influencing Knowledge of COVID-19 Prevention in Eastern Ethiopia

**DOI:** 10.1177/21582440241273871

**Published:** 2024-09-19

**Authors:** Merga Dheresa, Zachary J. Madewell, Jonathan A. Muir, Tamirat Getachew, Gamachis Daraje, Gezahegn Mengesha, Cynthia G. Whitney, Nega Assefa, Solveig A. Cunningham

**Affiliations:** 1 Haramaya University, Harar, Ethiopia; 2 Centers for Disease Control and Prevention, Atlanta, GA, USA; 3 Emory University, Atlanta, GA, USA

**Keywords:** community, household, SARS-CoV-2, sub-Saharan Africa, health education, health communication

## Abstract

This study examined coronavirus disease 2019 (COVID-19) prevention knowledge among community residents in Eastern Ethiopia to support public health interventions and vaccination coverage. A cross-sectional survey in August-September 2021 recruited 880 participants from households in a Health and Demographic Surveillance System (HDSS) in Harari and Oromia, Ethiopia. Participants were randomly selected and interviewed in person using tablets and a standardized questionnaire. Principal components analysis was used to create a score representing COVID-19 prevention knowledge. Quasi-Poisson regression was used to examine associations between demographic characteristics and COVID-19 prevention knowledge. The survey also assessed awareness of community/government COVID-19 prevention measures and healthcare services for under-5 children and pregnant women. The most cited COVID-19 prevention measures were handwashing with soap (91.5%) and wearing facemasks (89.2%); least mentioned were avoiding travel (22.2%) and wearing medical gloves (20.3%). Commonly recognized community/government measures included school closures (77.0%), avoiding gatherings (75.2%), and staying home (62.3%). Adjusted analyses demonstrated higher COVID-19 prevention knowledge among rural participants, those aged ⩾ 65 years (reference: <25), with secondary education (reference: no education), with monthly income of ⩾ 2,001 Birr (reference: 0–1,200), and were farmers, domestic/subsistence workers, or government employees (reference: unemployed). Knowledge was lower among households with ⩾ 5 household members. Of households with under-5 children and pregnant women, 9.4% and 12.3% missed medical care visits since mid-March 2020 consequent to the pandemic. Public health interventions to reduce COVID-19 transmission rely on risk perception and knowledge. Understanding these factors can help Ethiopian authorities design effective health education programs to control community and household SARS-CoV-2 transmission.

## Plain language summary

Understanding COVID-19 prevention in Eastern Ethiopia

This research evaluated knowledge of COVID-19 prevention in Eastern Ethiopia. We surveyed over 800 people from households in two cities. Most people knew about washing hands with soap and wearing masks, but fewer cited avoiding travel and unnecessary outings. Older adults, rural residents, and those with higher education and income had better knowledge about preventing COVID-19, whereas large families had lower knowledge. Some families missed medical visits for children and pregnant women because of the pandemic. This information can help health officials design better programs to teach people how to stay safe from COVID-19.

## Introduction

Coronavirus disease 2019 (COVID-19) was first reported in Ethiopia in March 2020. Since then, over 500,000 laboratory-confirmed cases and over 7,500 deaths have been reported in Ethiopia through May 2023, accounting for 5.2% of total COVID-19 cases and 4.3% of COVID-19 deaths reported in Africa (World Health Organization, 2022b). However, limited COVID-19 testing throughout the pandemic likely resulted in a significant underestimation of the true burden of the disease in Ethiopia. As of March 2022, only 3 million COVID-19 tests had been performed in the country of 120 million people, implying that the actual number of cases is likely much higher than reported figures (Konjit-Solomon, 2023).

Knowledge, attitude, and practices (KAP) studies in sub-Saharan Africa have demonstrated positive associations between knowledge of COVID-19 symptoms, transmission, and prevention, and the implementation of public health measures such as hand hygiene, social distancing, and facemask use (Nwagbara et al., 2021; Tshotetsi et al., 2023; Yesuf & Abdu, 2022). Furthermore, knowledge of COVID-19 symptoms, transmission, and prevention has been linked to higher vaccination coverage (Abebe et al., 2021; Aklil & Temesgan, 2022; Yisak et al., 2022), one of the most effective tools for protecting individuals from severe hospitalization and death (Shapiro et al., 2021; Song et al., 2023, 2024). COVID-19 vaccines were introduced in Ethiopia in March 2021, initially prioritizing healthcare workers, older adults, and individuals with chronic diseases (World Health Organization, 2021a). The vaccination campaign was expanded in November 2021 to include all individuals aged 12 years or older (WHO, 2021b). By May 2023, 32.6% of Ethiopia’s population had completed a primary COVID-19 vaccine series of BBIBP-CorV (Sinopharm, Beijing CNBG; 2 doses), BBV152 (Bharat Biotech’s Covaxin; 2 doses), Ad26.COV2.S (Janssen; 1 dose), or ChAdOx1-S (Covishield; 2 doses), but only 2.5% had received a booster dose (World Health Organization, 2022b).

A previous study in the Harari Region of Eastern Ethiopia (February 2021) identified a low perceived risk of COVID-19, with only 35.6% of participants intending to seek healthcare if symptomatic (Eyeberu et al., 2021). However, these data predate the emergence of more transmissible SARS-CoV-2 variants such as Delta and Omicron (Madewell et al., 2023; Madewell, Yang, et al., 2022). While the study found a higher risk perception in rural areas, it did not assess factors related to knowledge of COVID-19 prevention measures, awareness of community interventions, or healthcare access for vulnerable populations like children under 5 and pregnant women (Eyeberu et al., 2021). To address this knowledge gap, we conducted a community-based cross-sectional study in Eastern Ethiopia to evaluate factors associated with knowledge of COVID-19 prevention in both urban and rural settings. The findings from this study will inform the prioritization of health education programs designed to promote public health measures and ultimately prevent the transmission of infectious diseases within communities.

## Methods

### Study Setting

The study was conducted in a predominantly rural area in the Kersa District and an urban area in the Harari People’s National Regional State in Eastern Ethiopia. Both areas have been monitored through a Health and Demographic Surveillance System (HDSS), with the Kersa study site established in 2007 and the Harar site established in 2012, where demographic and health-related information has been regularly collected (Muir et al., 2023b; Muir, Dheresa, et al., 2024). The rural area comprises 24 kebeles (neighborhoods), covering 353 km^2^ and with a population of 135,754 in 25,653 households (Figure 1). The urban area comprises 12 kebeles, covering 25.4 km^2^ and with a population of 55,773 in 14,768 households.

### Study Design

We conducted a cross-sectional survey to assess knowledge of COVID-19 prevention measures among a sample of households in Eastern Ethiopia. This study is part of a larger examination within the Child Health and Mortality Prevention Surveillance (CHAMPS) network to understand the consequences of COVID-19 lockdowns for child health and mortality (Cunningham et al., 2019; Muir et al., 2023a, 2023b; Muir, Dheresa, et al., 2024; Muir, Matsena, et al., 2024; Muir, Onwuchekwa, et al., 2024; Nhacolo et al., 2023). A survey instrument was developed to collect information about households’ experiences during the COVID-19 lockdown, including their knowledge of personal and community or government measures to prevent COVID-19, access to healthcare services for under-5 children, and healthcare services for pregnant women.

Simple random sampling was used to select a sample of the households residing within the HDSS catchment areas. A target sample size was set to 440 households from Kersa and another 440 households from Harar (Muir et al., 2023a, 2023b; Muir, Dheresa, et al., 2024). These target sample sizes were achieved, resulting in a combined sample size of 880 households (Figure 1). Sample weights were not applied. The sample size was specified a priori to detect prevalence of changes in accessing healthcare, with specifications of 50% of the population experiencing changes, 95% confidence interval (CI), precision of 0.05, and non-response adjustment of 10%. Study participants comprised a qualifying representative (the household head or an adult household member with good knowledge about the household).

### Data Collection and Quality Assurance

Data collectors were selected from the fieldwork teams of HDSS enumerators, who were already trained and working in the HDSS. Data were collected through a standardized questionnaire administered by trained interviewers using tablets during in-person household visits between August and September 2021. Interviewers ensured participants understood the questions and accurately recorded their responses. All 880 sampled households consented and participated in the survey.

To ensure data quality, we developed the survey instrument following established methods (Muir et al., 2023a; Ruel et al., 2015). This process involved identifying key research questions and concepts and conducting a rapid review of relevant literature to inform question development. Additionally, we identified existing survey instruments, such as the World Bank’s “High Frequency Mobile Phone Surveys of Households to Assess the Impacts of COVID-19,” as a reference point from which a subset of survey questions was selected and revised. A standardized survey instrument, originally prepared in English for implementation across sites within the CHAMPS network, was adapted to increase its suitability with local cultural and social contexts in Harari, Ethiopia. The data collection instrument did not formally seek to obtain ascertainment of COVID-19 cases in the household, timing, and case definition; COVID-19 lab tests were not included as part of the data collection. Data collection was carried out using the RedCAP platform for electronic data collection, which included translations of the English version of the instrument into Amharic and Afaan Oromo (Muir et al., 2023a).

Data from the questionnaire were linked with data from the most recent completed HDSS round (collected from January to May 2020) to incorporate additional demographic data about the sampled household, specifically: number of children under 5 years of age, number of adults over age 60 in the household, household assets, and residence construction materials. Data quality assurance and cleaning followed standard procedures for the HDSS (Assefa et al., 2016; Cunningham & Muir, 2023), and inconsistent or missing data were flagged for data collectors to correct. Data quality control included pretesting the survey instrument on a sub-sample of respondents who were not eligible for the study (Muir et al., 2023a). Findings and experiences from the pretest were utilized in revising the research data collection tools. Data collectors and supervisors were provided training concerning the objective of the study, confidentiality of information, and techniques of data collection. Field supervisors and the field coordinator selected a random sub-sample of questionnaires for re-visits to validate the recorded information.

### Measures

Interviewers asked which measures can be adopted to reduce the risk of contracting COVID-19 without providing options and recorded participants’ responses. Responses were subsequently categorized as: handwashing, sanitizer use, avoiding handshaking/physical greeting, mask use, medical gloves use, avoiding travel, avoiding going out, avoiding crowded places, 2 m social distancing, and other.

This survey included a closed-ended question asking participants which steps the community or government had taken to prevent the spread of SARS-CoV-2. Interviewers read each of the following options aloud and recorded participants’ responses: advising citizens to stay home, avoiding gatherings, restricting travel within country/area, restricting international travel, closing of schools and universities, imposing curfew or lockdown, closing of non-essential businesses, conducting sensitization or public awareness campaigns, establishing isolation centers, and disinfecting of public places.

Individual characteristics included: sex (female, male), age group (<25, 25–44, 45–64, ⩾ 65 years), residence (urban, rural), ethnicity (Oromo, Amhara, other), religion (Christian, Muslim, other), marital status (married, separated/divorced, widowed, single), education (no formal education, primary, secondary, college), occupation (unemployed, farmer or domestic/subsistence worker, government employee, private employee, farmer, other), and health insurance (yes, no). Household variables were: household size (1–2, 3–4, 5–6, 7–8, ⩾ 9), children under 5 years (yes, no), adults over 60 years (yes, no), pregnant women (yes, no), household member tested positive for SARS-CoV-2 (yes, no), monthly income (⩾ 4,600, 3,001–4,600, 2,001–3,000, 1,201–2,000, 0–1,200 Birr, which is roughly equivalent in U.S. dollars to ⩾ $127, $83–127, $55–83, $33–55, and $ 0–33 ($1 USD ≈ 36.4 Birr at the time of this survey in August to September 2021), and television ownership (yes, no).

For participants with children under 5, our survey asked whether those children had attended any healthcare visits between mid-March 2020 (yes, no) and when the survey was conducted in August to September 2021, and whether they needed medical care or a clinic visit but could not do so since mid-March 2020 (yes, no). We recorded the kind of medical care the child received or needed but did not receive (routine follow-up visits, routine vaccinations, clinic visits for any illness, services for malnutrition), the number of missed medical care or clinic visits, and reasons for not receiving healthcare (clinic closed, out of vaccines or medications, did not get transportation, lockdown, afraid to go).

For households with pregnant women between mid-March 2020 and August to September 2021, we asked whether they had attended any pregnancy-related healthcare since mid-March (yes, no) and whether they needed medical care during pregnancy but did not receive it (yes, no). We also recorded the kind of healthcare received or needed but not received (routine antenatal care visits, pregnancy-related complication or concern, delivery, Cesarean section, illness not related to pregnancy, medications, routine postanal care visit, postnatal concern or complications), the number of missed medical care or clinic visits, and reasons for not receiving healthcare (out of medications, lockdown, afraid to go).

### Statistical Analysis

We presented frequency distributions of individual characteristics (age, sex, residence, ethnicity, religion, marital status, education, occupation, has health insurance), household characteristics (household size, children under 5, adults over 60, pregnant women, income, television, household member tested positive for SARS-CoV-2), child healthcare access (had children under 5 attend healthcare visits, needed medical care but could not do so, number of visits missed, reasons for not receiving healthcare), and pregnancy healthcare access (attended pregnancy-related healthcare, needed medical care but could not get it, number of visits missed, reasons for not receiving healthcare). We reported frequencies and 95% confidence intervals (95% CI) for knowledge of individual and community or government measures to prevent COVID-19. Given the differences in COVID-19 knowledge reported by urban and rural residents (Eyeberu et al., 2021), we presented all results for the total population and stratified by urban/rural residence. Pearson Chi-square tests were used to evaluate associations between demographic characteristics and urban or rural residence.

In accord with other KAP studies (Hajj et al., 2022; Kwabla et al., 2022; Madewell, Chacón-Fuentes, et al., 2022; Madewell, Chacón-Fuentes, Jara, Mejía-Santos, Molina, Alvis-Estrada, Ortiz et al., 2021; Nhacolo et al., 2023), we used principal components analysis (PCA) to create a score for assessing the level of knowledge of COVID-19 prevention among all prevention variables, excluding gloves use, which may offer limited protection against SARS-CoV-2 transmission among community members (Anedda et al., 2020). The overall Kaiser-Meyer-Olkin index of sampling adequacy was 0.89, indicating that the sample size and data were sufficient for PCA. Scores of one were assigned to an option if a respondent mentioned it and zero if they did not mention it. The resulting compound factor accounted for 46% of the variability in the data and included all variables (Supplemental Table S1). These variables were then weighted against their eigenvector coefficients. Knowledge of prevention scores ranged from 0 to 4.5, with higher scores representing greater knowledge.

Quasi-Poisson regression was used to evaluate associations between characteristics (age group, sex, residence, ethnicity, religion, marital status, education, occupation, monthly income, health insurance, household size, children under 5, adults over 60, pregnant women, and television as independent variables) and PCA-derived knowledge of prevention index (dependent variable) for (1) all participants and (2) stratified by urban/rural residence. Unadjusted analyses were first examined, followed by an adjusted model considering all characteristics simultaneously. Variables were selected for inclusion in the final adjusted regression models if their unadjusted associations with knowledge of COVID-19 prevention had theoretical justification. Other studies have demonstrated significant associations between COVID-19 knowledge and age (Bitew et al., 2021; Nhacolo et al., 2023; Sulistyawati et al., 2021); sex (Bitew et al., 2021; Nhacolo et al., 2023); ethnicity (Nhacolo et al., 2023); religion (Nhacolo et al., 2023); marital status (Bitew et al., 2021; Nhacolo et al., 2023); education (Nhacolo et al., 2023; Yesse et al., 2021); occupation (Defar et al., 2021; Nhacolo et al., 2023); income (Nhacolo et al., 2023); health insurance (Kreps et al., 2020); household size (Feleke et al., 2022; Taddese et al., 2021); and television as a source of COVID-19 information (Nhacolo et al., 2023). Therefore, all variables from unadjusted analyses were included in the final model. Values of *p* < .05 were considered statistically significant. We checked for multicollinearity using generalized variance inflation factors (GVIF) among all independent variables (all GVIFs were <1.7) (Supplemental Table S2) and evaluated residual deviance as a goodness-of-fit test for the overall model (residual deviance = 381.3, *p* = 1.0). All analyses were done in R software, version 4.3.0 (R Foundation for Statistical Computing, Vienna, Austria).

## Results

Of all 880 participants, 81.9% were female, 65.7% identified as Oromo ethnicity, 69.1% were Muslim, 67.0% were married, and 18.8% widowed. Half (50.2%) had completed at least primary education, and 42.1% had health insurance. Regarding households, 43.8% had children under 5, 26.8% had adults over 60, and 13.0% had pregnant women (Table 1). Urban participants were older (median age 45 years; IQR: 35–60) than rural participants (median age 39 years; IQR: 30–48) (*p* < .001). Two hundred sixty-six participants (30.2%) had family members tested for COVID-19, with 9.0% reporting positive cases. Compared to urban residents, a larger proportion of rural residents were Oromo (92.5% vs. 38.9%), Muslim (90.5% vs. 47.7%), married (79.1% vs. 55.0%), lacked formal education (72.3% vs. 27.3%), and worked in agriculture or domestic roles (53.0% vs. 4.5%; *p* ⩽ .035). The median household size in rural Kersa (6, IQR: 4–7) was larger than urban Harar (4, IQR: 3–5; *p* < .001). Rural households were larger (median size 6 vs. 4) and had more young children and pregnant women but fewer older adults and COVID-19 cases (*p* ⩽ .006). Urban dwellers had higher TV ownership (87.3% vs. 19.3%) and income (median 3,000 Birr vs. 2,000 Birr; *p* < .001).

Nearly all participants (99.3%, 874/880) were aware of COVID-19. Commonly cited community or government measures to prevent SARS-CoV-2 transmission were school closures (77.0%), avoiding gatherings (75.2%), and staying home (62.3%), whereas less mentioned were international travel restrictions (42.5%), curfews/lockdowns (43.0%), and establishment of isolation centers (43.5%) (Supplemental Figure S1). Urban participants more frequently mentioned school closures, staying home, and public awareness campaigns compared to rural participants (*p* < .001) (Figure 2).

The most mentioned individual measures to reduce the risk of contracting COVID-19 were handwashing with soap (91.5%), wearing facemasks (89.2%), and using hand sanitizer (67.2%), whereas fewer mentioned wearing medical gloves (20.3%), avoiding travel (22.2%), and unnecessary outings (23.1%) (Supplemental Figure S1). Rural residents more frequently cited avoiding outings (44.1% vs. 2.0%), travel (41.6% vs. 2.7%), and crowded places (66.8% vs. 28.4%) compared to urban residents (*p* < .001; Figure 2). Rural participants more frequently cited handwashing, mask use, social distancing, avoiding physical contact, and wearing gloves than urban participants, whereas fewer mentioned using hand sanitizers (*p* ⩽ .001).

Unadjusted analyses between demographic characteristics and PCA-derived knowledge of prevention overall for all participants and stratified by urban/rural residence are shown in Figure 3 and Supplemental Figure S2, respectively. Adjusting for other variables (Figure 3), urban participants had a lower COVID-19 prevention knowledge score (−0.77, 95% CI: [−0.86, −0.69]) compared to rural participants. Factors positively associated with COVID-19 prevention knowledge included higher monthly income levels (β: .13–.67), older age (β: .38), working in agriculture/domestic roles or as government employees (β: .24 and .14, respectively), being Christian (β: .16), being single (β: .18), and having secondary education (β: .17). Conversely, larger household sizes were associated with lower COVID-19 knowledge (β: −.29 to −.18). These associations were generally consistent across urban and rural groups, although certain associations like older age and household size were significant only for rural residents (*p* ⩽ .001) (Supplemental Figure S3). Furthermore, secondary education and Christian religion were only positively associated with COVID-19 knowledge for participants residing in urban areas (*p* ⩽ .029).

Over half (59.0%, 227/385) of households with children under 5 attended child healthcare services between mid-March 2020 and August-September 2021, mainly for routine or follow-up visits (41.9%, 95/227), illnesses (41.4%, 94/227), and vaccinations (33.9%, 77/227) (Table 2). However, 9.4% (36/385) of households needed medical care during this time but did not receive it. Reasons for not receiving healthcare services were fear of going to the clinic (61.1%, 22/36), lockdown restrictions (22.2%, 8/36), and vaccine or medication shortages (19.4%, 7/385); only three (0.8%) did not receive care because the clinic was closed. The primary missed care involved vaccinations (63.9%) and clinic visits for illnesses (41.7%), with a median of 2 missed visits (IQR: 1–3).

Almost three-quarters (73.7%, 84/114) of households with pregnant women had a pregnancy-related healthcare visit between mid-March 2020 and August-September 2021, mainly for pregnancy-related complications or concerns (75.0%, 63/84), routine antenatal care visits (47.6%, 40/84), and delivery (32.1%, 27/84) (Table 3). Fourteen (12.3%) pregnant women required medical care during pregnancy since mid-March 2020 but did not receive it, primarily due to pregnancy-related complications (42.9%, 6/14) and routine antenatal care visits (28.6%, 4/14). The median number of missed visits was 1 (IQR: 1–2).

## Discussion

In this cross-sectional survey of community members in Eastern Ethiopia, 9 of 10 participants cited handwashing and facemask use as personal measures to prevent COVID-19, consistent with other studies conducted in Ethiopia (Bitew et al., 2021; Desalegn et al., 2021; Eyeberu et al., 2021). These measures have been shown in other settings to be associated with a substantial reduction in COVID-19 transmission (Talic et al., 2021). Conversely, medical glove use was the least mentioned measure, which aligns with public health guidance as COVID-19 primarily spreads through respiratory droplets, while fomite transmission is considered low-risk (Meister et al., 2022). The World Health Organization has warned that gloves use may have limited protective effectiveness for community members against SARS-CoV-2 (Anedda et al., 2020; World Health Organization, 2022a). Regular use of gloves may provide a false sense of protection, and their incorrect use may favor SARS-CoV-2 transmission (Anedda et al., 2020).

Participants from rural areas were significantly more likely than those from urban areas to cite social distancing, avoiding crowded places, traveling, going out, and physical greetings to prevent COVID-19, which are among the most effective ways to prevent SARS-CoV-2 transmission (Madewell et al., 2020; Talic et al., 2021). These findings align with a study conducted in Harari, which demonstrated that individuals residing in rural areas were more likely to adhere to COVID-19 prevention measures, including handwashing, staying at home, maintaining social distancing, and wearing a facemask (Eyeberu et al., 2022). This may reflect the greater feasibility of social distancing in rural settings with lower population density. Conversely, sanitizer use was higher in urban settings, possibly due to differences in access and availability, socioeconomic factors, public health messaging and awareness campaigns, or hygiene practices (Vuppu et al., 2023).

Television ownership was significantly lower in rural (19%) than urban (87%) areas, suggesting a potential disparity in information access. However, a higher proportion of rural residents had community health insurance (59% vs. 25% urban), indicating they might rely more on local healthcare professionals for information. Although previous studies in Ethiopia linked television, social media, and radio to greater knowledge of COVID-19 transmission and prevention (Bitew et al., 2021; Eyeberu et al., 2021; Yesuf & Abdu, 2022), our study did not find an association with TV ownership. Simply having a television does not guarantee exposure to accurate information; program content, viewing habits, and media literacy likely matter. Additional studies are needed to explore other influences on knowledge in the Harari Region, such as health infrastructure, cultural factors, and preferred communication channels.

Our study found higher COVID-19 prevention knowledge among participants aged 65 and older, aligning with previous research (Defar et al., 2021). This may be due to greater awareness of their vulnerability, as older adults face higher risks for severe COVID-19 outcomes (Goldstein & Lee, 2020; Wong, 2023). Other studies in Ethiopia also report increased vaccination and prevention measures among older adults (Abebe et al., 2021; Bitew et al., 2021). Although younger adults are less likely to be hospitalized with COVID-19 compared to older adults, some develop severe disease, and they can transmit the virus even when asymptomatic (Ravindra et al., 2022). Targeted interventions are needed to address potential knowledge gaps and mitigate transmission risks in younger age groups. Our study population’s higher median age (40 years) compared to Ethiopia’s young population (19.8 years) may suggest the observed knowledge advantage in older adults may not generalize to younger cohorts (Central Intelligence Agency, 2023).

Higher-income households generally had better COVID-19 knowledge, potentially due to greater access to information sources like media or the internet (Bitew et al., 2021; Yesuf & Abdu, 2022). However, in rural areas, household size was inversely associated with COVID-19 prevention knowledge, contrasting findings from urban studies (Taddese et al., 2021). In our rural sample, participants from households with nine or more members had notably lower rates of secondary or college education (0%) compared to smaller households (36.5% with secondary or college education), indicating a potential influence of limited education access on COVID-19 knowledge (Getawa et al., 2022).

Our study also found that individuals working in agriculture/domestic roles, government jobs, and those who identified as Christian demonstrated higher knowledge of COVID-19 prevention measures. While the reasons for these associations require further investigation, they might be related to factors like access to information, exposure to hygiene practices in the workplace, or faith-based messaging. Public health interventions could leverage these groups for knowledge dissemination by collaborating with religious leaders and tailoring communication materials for specific occupations.

In April 2020, Ethiopia declared a 5-month state of emergency for COVID-19, implementing measures such as suspending public gatherings, quarantining travelers, mandating masking, closing schools and universities, and ordering remote work (Huluka et al., 2022; Mohammed et al., 2020). Other actions included restricting taxi and mass transit services, restricting long-distance travel to and from Addis Ababa, closing borders, disseminating COVID-19 information via various media, establishing dedicated healthcare facilities, and repurposing non-healthcare sites as isolation centers and hospitals (Huluka et al., 2022; Mohammed et al., 2020). In the Harari Region, we found that the most recognized preventive measures were school closures, advice against gatherings, and urban residents’ awareness of government sensitization campaigns and stay-at-home advice, possibly influenced by city-based multimedia messaging (Bitew et al., 2021).

Our study also evaluated healthcare access for young children and pregnant women following the first COVID-19 case in Ethiopia in March 2020. Between mid-March and August to September 2021, over half of participants with children under 5 and three-quarters of pregnant women attended healthcare visits, but 1 in 10 missed visits due to COVID-19 concerns. These findings are consistent with a study in Southwest Ethiopia reporting reduced family planning, antenatal care visits, healthcare facility births, and newborn vaccinations during the pandemic (Kassie et al., 2021). During the pandemic in 2020 and 2021, there was a notable decline in childhood vaccination coverage, particularly in low and middle-income countries (SeyedAlinaghi et al., 2022). Ethiopia also experienced declines in childhood vaccination coverage and postponed measles preventive campaigns due to the pandemic (Nigus et al., 2020). Mitigating the pandemic’s impact on healthcare access requires addressing parental concerns, improving vaccine availability, reducing wait times at health centers, and promoting access in remote areas (Madewell, Jara, a-Santos, Molina, Alvis-Estrada, Coello-Licona et al., 2021; SeyedAlinaghi et al., 2022).

This cross-sectional study has limitations including the inability to establish causal relationships or temporality and potential social desirability bias in responses regarding COVID-19 prevention knowledge. We also acknowledge the possibility of interviewer bias, despite proactive measures such as interviewer training, rigorous recruitment, and supervision. Nonetheless, our study included a large sample of urban and rural community members, which provided the power to examine the data in several ways.

## Conclusion

In Harar and Kersa, Ethiopia, 9 of 10 community members knew that handwashing and mask-wearing could prevent COVID-19, but fewer identified avoiding crowded places and social distancing as prevention measures. Rural residents demonstrated better COVID-19 prevention knowledge than urban residents despite higher television ownership in urban areas, which may contribute to misinformation. These findings highlight the need for targeted outreach and messaging for younger, unemployed, and less educated individuals. Additionally, promoting vaccine safety and efficacy through tailored communication strategies involving community leaders is necessary. Future research should explore media impact, effective messaging strategies, vaccine acceptance, and long-term behavior change for better infectious disease control in communities.

## Supplementary Material

Supplementary Material

Supplemental material for this article is available online.

## Figures and Tables

**Figure 1.**
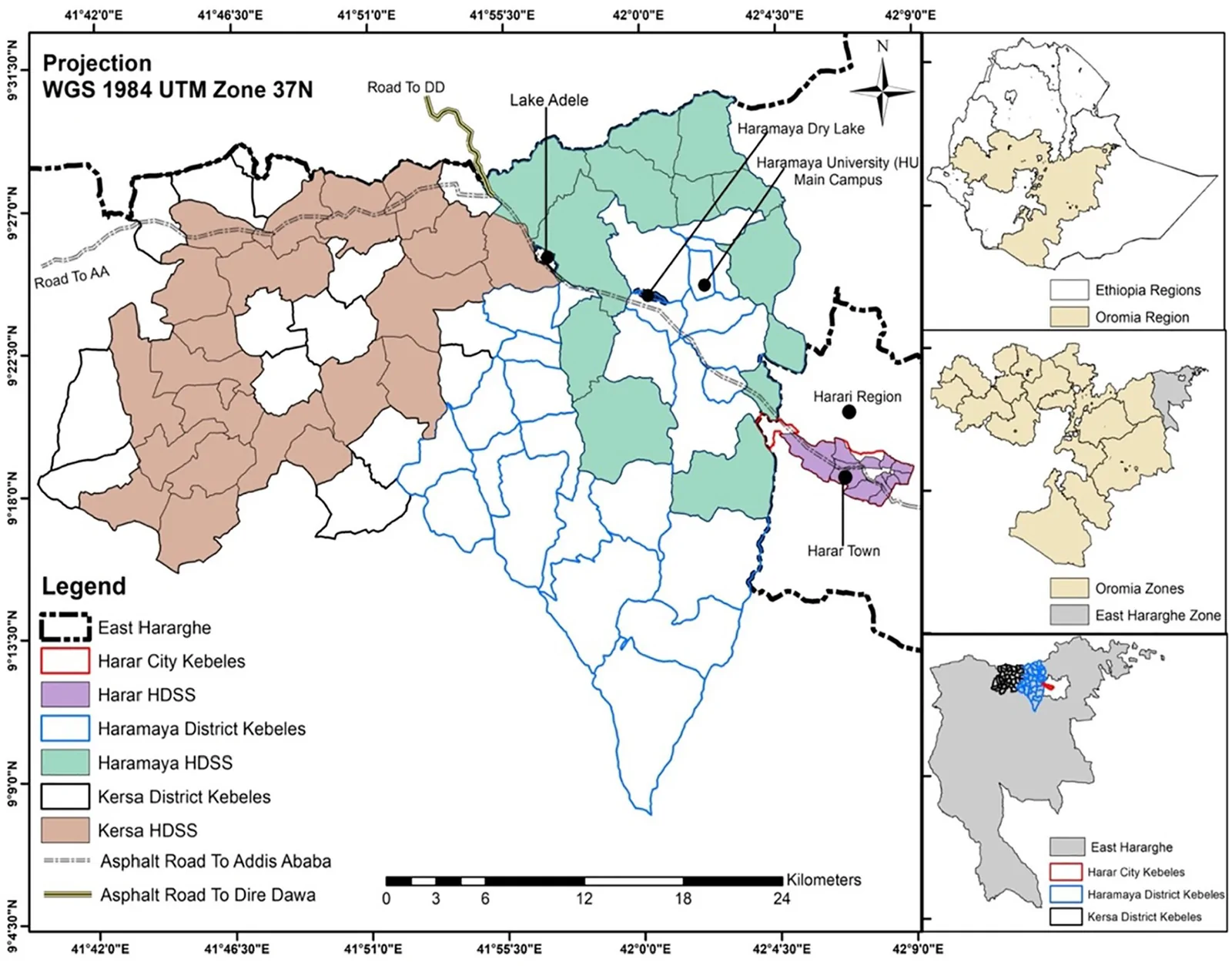
Geospatial distribution of the Harar and Kersa Health and Demographic Surveillance Systems (HDSS) within East Hararghe, Oromia, Ethiopia. The smaller map panels on the right identify the location of the HDSS catchment areas within the East Haraghe Zone of the Oromia region in Ethiopia. The HDSS catchment in Haramaya (depicted in green) was in development during the data collection period, so households from this catchment were not included in this study.

**Figure 2.**
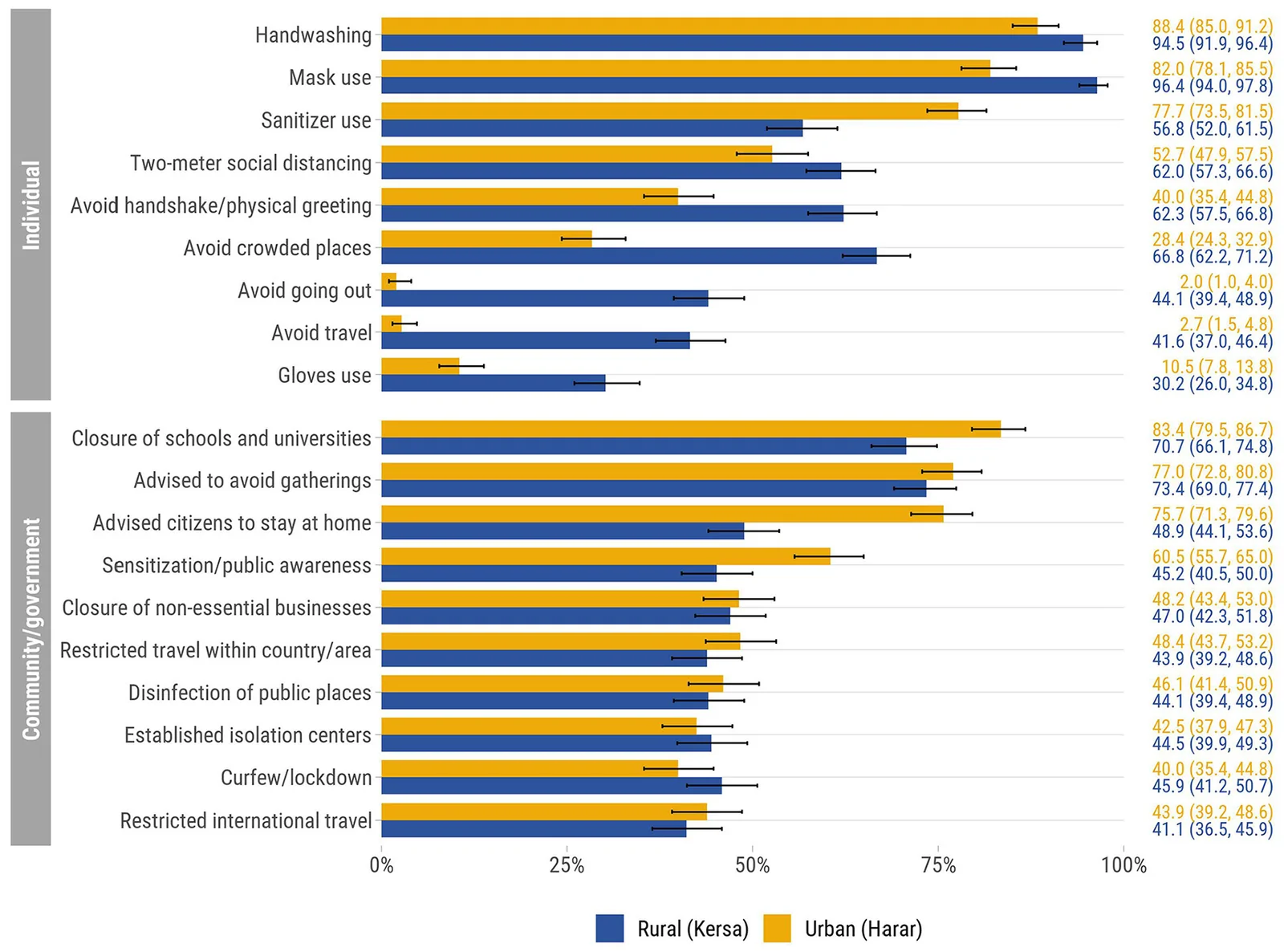
Knowledge of individual and community/government measures to prevent COVID-19 stratified by urban (Harar) and rural (Kersa) residence, Ethiopia, August to September 2021 (*N* = 880). Error bars represent 95% confidence intervals.

**Figure 3.**
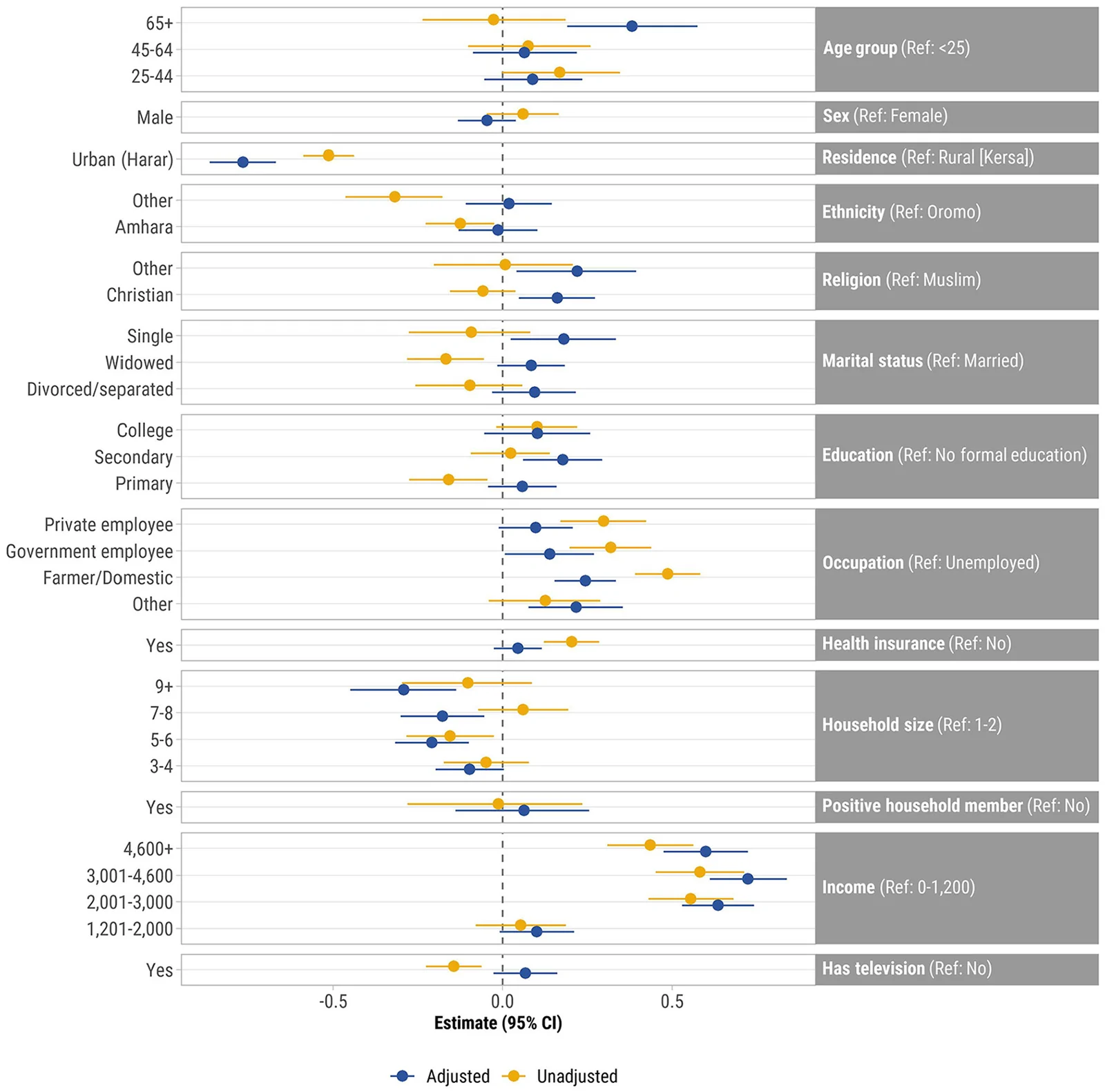
Unadjusted and adjusted associations between demographic characteristics and knowledge of COVID-19 prevention index, all participants (*N* = 880), Ethiopia, August to September 2021. Points represent β coefficients and error bars represent 95% confidence intervals. Adjusted for all other variables in the model. Knowledge of prevention was derived from principal components analysis and includes: handwashing, sanitizer use, mask wearing, avoiding handshaking/physical greeting, avoiding travel, avoiding going out, avoiding crowded places, and 2 m social distancing.

**Table 1.** Descriptive Statistics of Individuals Who Participated in COVID-19 Cross-Sectional Survey, Ethiopia, August to September 2021, (*N* = 880).

Characteristic	Total, *N* = 880	Urban, *N* = 440	Rural, *N* = 440	*p*-Value
	*N* (%)	*N* (%)	*N* (%)	
Individual characteristics				
Age (years)				<.001
<25	63 (7.2)	30 (6.8)	33 (7.5)	
25–44	437 (49.7)	184 (41.8)	253 (57.5)	
45–64	280 (31.8)	146 (33.2)	134 (30.5)	
⩾65	100 (11.3)	80 (18.2)	20 (4.5)	
Female sex	721 (81.9)	348 (79.1)	373 (84.8)	.035
Ethnicity				<.001
Oromo	578 (65.7)	171 (38.9)	407 (92.5)	
Amhara	198 (22.5)	165 (37.5)	33 (7.5)	
Other	104 (11.8)	104 (23.6)	0 (0.0)	
Religion				<.001
Orthodox	234 (26.6)	197 (44.8)	37 (8.4)	
Muslim	608 (69.1)	210 (47.7)	398 (90.5)	
Other	38 (4.3)	33 (7.5)	5 (1.1)	
Marital status				<.001
Married	590 (67.0)	242 (55.0)	348 (79.1)	
Divorced/separated	71 (8.1)	45 (10.2)	26 (5.9)	
Widowed	165 (18.8)	112 (25.5)	53 (12.0)	
Single	54 (6.1)	41 (9.3)	13 (3.0)	
Education				<.001
No formal education	438 (49.8)	120 (27.3)	318 (72.3)	
Primary	176 (20.0)	118 (26.8)	58 (13.2)	
Secondary	143 (16.2)	109 (24.8)	34 (7.7)	
College	123 (14.0)	93 (21.1)	30 (6.8)	
Occupation				<.001
Unemployed	315 (35.8)	192 (43.6)	123 (28.0)	
Government employee	131 (14.9)	104 (23.6)	27 (6.1)	
Private employee	114 (13.0)	62 (14.1)	52 (11.8)	
Farmer/Domestic	253 (28.7)	20 (4.5)	233 (53.0)	
Other	67 (7.6)	62 (14.1)	5 (1.1)	
Has health insurance	370 (42.1)	111 (25.2)	259 (58.9)	
Household characteristics				
Household size				<.001
1–2	146 (16.6)	94 (21.4)	52 (11.8)	
3–4	257 (29.2)	168 (38.2)	89 (20.2)	
5–6	247 (28.1)	122 (27.7)	125 (28.4)	
7–8	171 (19.4)	37 (8.4)	134 (30.5)	
⩾9	59 (6.7)	19 (4.3)	40 (9.1)	
Children under 5 years	385 (43.8)	129 (29.3)	256 (58.2)	<.001
Adults over 60 years	236 (26.8)	134 (30.5)	102 (23.2)	.006
Pregnant women	114 (13.0)	40 (9.1)	74 (16.8)	<.001
Household member tested positive for SARS-CoV-2	24 (2.7)	20 (4.5)	4 (0.9)	.001
Income				<.001
⩾4,600	175 (19.9)	96 (21.8)	97 (22.0)	
3,001–4,600	134 (15.2)	73 (16.6)	137 (31.1)	
2,001–3,000	168 (19.1)	75 (17.0)	93 (21.1)	
1,201–2,000	210 (23.9)	71 (16.1)	63 (14.3)	
0–1,200	193 (21.9)	125 (28.4)	50 (11.4)	
Owns television	469 (53.3)	384 (87.3)	85 (19.3)	<.001

**Table 2.** Child Healthcare Access, Ethiopia, August to September 2021 (*N* = 385).

Child healthcare access	*N*	%
Had children under 5 attend any healthcare	227	59.0
visits since mid-March 2020		
Routine follow-up visit	95	24.7
Childhood vaccinations	77	20.0
Clinic visits for any illness	94	24.4
Services for malnutrition	8	2.1
Needed medical care/clinic visit but could not do so since mid-March 2020	36	9.4
Routine follow-up visit	7	1.8
Routine vaccinations	23	6.0
Clinic visits for any illness	15	3.9
Services for malnutrition	2	0.5
Number of medical care/clinic visits missed		
1	9	2.3
2	11	2.9
⩾3	6	4.2
Reasons for not receiving healthcare^a^		
Clinic closed	3	0.8
Out of vaccines or medications	7	1.8
Did not get transportation	5	1.3
Lockdown	8	2.1
Afraid to go	22	5.7
No response	1	0.3

a Participants could provide multiple reasons.

**Table 3.** Pregnancy Healthcare Access, Ethiopia, August to September 2021 (*N* = 114).

Pregnancy healthcare access	*N*	%
Attended any pregnancy-related healthcare since mid-March 2020	84	73.7
Routine antenatal care visits	40	35.1
Pregnancy-related complication or concern	63	55.3
Delivery	27	23.7
Caesarean section	3	3.5
Illness not related to pregnancy	9	7.9
Medications	2	1.8
Routine postnatal care visit	6	5.3
Postnatal concern or complications	1	0.9
Needed medical care during pregnancy but could not do so since mid-March 2020	14	12.3
Routine antenatal care visits	4	3.5
Pregnancy-related complication or concern	6	5.3
Illness not related to pregnancy	2	1.8
Postnatal concern or complications	2	1.8
Number of medical care/clinic visits missed		
1	6	5.3
2	4	3.5
Reasons for not receiving healthcare		
Out of medications	1	0.9
Lockdown	1	0.9
Afraid to go	1	0.9
No response	11	78.6

## Data Availability

Repository name: CHAMPS Population Surveillance Dataverse Data identification number: https://doi.org/10.15139/S3/CZO1IX Direct URL to data: https://dataverse.unc.edu/dataset.xhtml?persistentId=doi:10.15139/S3/CZO1IX Instructions for accessing these data: A deidentified version of the data are published and publicly available through the CHAMPS Population Surveillance Dataverse.
